# Intestinal angioleiomyoma in a dog

**DOI:** 10.1093/jvimsj/aalag281

**Published:** 2026-10-27

**Authors:** Ran Nivy, Yaron Bruchim, Thomas Eley

**Affiliations:** Koret School of Veterinary Medicine, The Hebrew University of Jerusalem, P.O. Box 12, Rehovot 761001, Israel; Ben-Shemen Specialist Referral Center, Ben-Shemen Youth Village, Israel; Ben-Shemen Specialist Referral Center, Ben-Shemen Youth Village, Israel; IDEXX Laboratories Ltd. (pathology services), Grange House, Sandbeck Way, Wetherby LS22 7DN, United Kingdom

**Keywords:** anemia, blood loss, intussusception, iron deficiency, microcytosis

## Abstract

Angioleiomyoma is a rare, smooth muscle tumor of vascular origin, which primarily affects the skin. A 9-year-old, castrated male dog presented for transient ataxia. Abnormalities were not detected on physical examination, but blood tests revealed a mild, severely microcytic anemia and transferrin saturation of 2.5% (reference interval, 27%-66%). An exhaustive diagnostic work-up ensued but failed to find the cause of the severe iron deficiency in laboratory, imaging, and endoscopic studies. Severely microcytic anemia had persisted for 6 months, despite iron-dextran injections, and in the absence of clinical signs, until intussusception developed. The abnormal jejunal segment was removed and diagnosed as angioleiomyoma, based on characteristic histological features, positive immunolabeling with α-smooth muscle actin and CD31, and negative S100 and c-Kit/CD117 staining. Hitherto unreported in dogs, intestinal angioleiomyoma was associated with protracted, occult, asymptomatic intestinal blood loss, until intussusception developed. Prognosis appears favorable with full excision, based on clinical behavior in other organs, and the present case.

## Introduction

A 9-year-old, castrated male mixed-breed dog presented with a chief complaint of acute hindlimb weakness and unsteady gait. The dog had no important medical history, was regularly vaccinated with core vaccines, and prophylactically dewormed every 3 months. The day before presentation, the dog exhibited transient difficulty in rising from sternal recumbency, hindlimb paresis and ataxia. The dog had fully recovered after several minutes and was taken to the referring veterinarian shortly thereafter, where neither weakness nor any ambulatory impairment was appreciated. Nevertheless, the next day the dog was referred for further diagnostic work-up. Upon presentation, the dog was bright, alert, and responsive, had a body condition score of 4/9, with a pulse of 100 beats/min, a respiratory rate of 24 breaths/min, and a rectal temperature of 37.8°C. Abnormal findings included mildly pale mucous membranes and a grade 2/6, band-shaped, holosystolic, left apical heart murmur. There was no evidence of hematochezia or melena in the feces during rectal exam. Ectoparasites were not identified either. Blood tests, including CBC and chemistry, only revealed hypochromic, markedly microcytic anemia (Table S1). In the blood smear, mild polychromasia, anisocytosis, and spherocytosis were noted. Eosinophilia was absent, neutrophils were mature and nontoxic, and an in-saline slide agglutination test at room temperature was negative. No pathologies were noted in chest x-rays and there was no discernible esophageal pathology. Subtle, nonspecific changes were noted during abdominal ultrasonography, performed by a European College of Veterinary Diagnostic Imaging (ECVDI)-certified radiologist, and comprised a mildly small liver which was smoothly marginated with a slightly coarse parenchyma diffusely, and one jejunal segment which showed mucosal speckling, but no pathological changes in wall thickness or layering. The dog received iron (iron dextran, 10 mg/kg, IM, Fatro S.p.A) and omeprazole (1.2 mg/kg, PO, q12h, Dexcel Pharma) and returned a month later for evaluation by a certified internist (R.N., first author). During this month, barring 2, several-minute-long episodes of weakness as previously described, the dog exhibited no clinical signs. There were no findings on physical exam, and no heart murmur. The CBC revealed mild anemia with marked microcytosis (Table S1), and mild polychromasia, anisocytosis, and hypochromasia in the blood smear. Additional tests included prothrombin and partial thromboplastin times, vitamin B12 and folic acid concentrations (all tests were within their respective reference intervals), bile acids (pre- and post-prandial measurements), and serum iron and unsaturated iron-binding capacity (UIBC) concentrations (Table S1). Overt and microscopic urinary bleeding was ruled out using a urine sample obtained by cystocentesis. The combination of low serum iron concentration, an elevated UIBC concentration and a markedly low transferrin saturation corroborated the clinical suspicion of iron deficiency. Alternative differentials for such microcytosis, including breed-related microcytosis, drug/toxin exposure, and portosystemic shunts were ruled out.^1^ However, no indication of external or internal blood loss could be identified. Since gastrointestinal blood loss is one of the most common causes of iron deficiency in dogs,^1^ endoscopy was performed, with no evidence of a bleeding lesion throughout the esophagus, stomach, and duodenum. The dog recovered uneventfully from the procedure, received a third injection of iron dextran, and was discharged with instructions to continue iron injections twice monthly if anemia and microcytosis persist, in addition to omeprazole treatment. Histology results from the stomach and duodenum revealed a nonspecific, multifocal, mild, and chronic lymphoplasmacytic and eosinophilic gastritis and nonspecific, multifocal moderate, and chronic lymphoplasmacytic to eosinophilic duodenitis. A PCR test for antigen receptor rearrangement (PARR) analysis demonstrated polyclonal rearrangements of antigen receptor genes in both T-cell and B-cell populations, thereby further lowering the suspicion of gastrointestinal lymphoma. A video capsule endoscopy procedure had been offered, in search of bleeding lesions along more distal, endoscopically inaccessible parts of the intestinal tract, which the owners declined. Six months had elapsed before the dog presented again. According to his owners, the dog’s appetite and activity had remained unchanged until 3 weeks before presentation, when daily episodes of vomiting and watery diarrhea appeared. Vomiting decreased in frequency with metoclopramide treatment, prescribed by the referring veterinarian, but diarrhea persisted to variable degrees. Notwithstanding a reportedly healthy appetite, the dog had lost 4 kg (15% of the original body weight). Blood tests were reminiscent of those performed six months earlier, with severely microcytic anemia being the only salient abnormality (Table S1). Mild leukocytosis, the result of mature neutrophilia, was similar in magnitude to the first visit, and believed to be a nonspecific response to an underlying disease associated with the persistent microcytic anemia. An additional abdominal ultrasonography scan was performed, revealing a long segment of small intestinal intussusception in mid-abdomen. Subsequently, the dog underwent laparotomy, wherein a mid-jejunal, 20-cm-long intussusception was identified. Proximal to the intussusception the entire small intestines were thickened with prominent lymphatics, while distally the rest of the intestinal tract was normal. Manual reduction of the intussusception failed owing to excessive tissue friability, and the entire segment was removed. No discernible mass was noted by the surgeon within the excised, intussuscepted segment. Additional biopsies were obtained from the liver, which appeared reduced in size, albeit with normal gross appearance and color, and from mesenteric lymph-nodes, notwithstanding their normal size.

Histologically, adjacent to the central area of intussusception, the muscularis and serosa were expanded by a well-demarcated, unencapsulated spindle cell proliferation composed of loosely arranged cells. These contained a large amount of eosinophilic cytoplasm an oval nucleus with finely stippled chromatin, and a single small amphophilic nucleolus. The neoplastic cells were diffusely separated by large numbers of thin-walled capillaries, and fewer larger irregular vascular channels, whose endothelium exhibited no atypia (Figure 1A). Antigen retrieval was performed using heat-induced epitope retrieval, and sections were treated at full pressure with Access Retrieval Unit (Menarini) in sodium citrate buffer (pH 6) for smooth muscle actin (SMA), EDTA buffer (pH = 9) for CD117 and CD31 for 90 s at 125°C. The sections were then rinsed in Tris Tween buffer (pH = 7.5). Antibody dilutions used were SMA 1:100 (Dako, M0851), CD117 1:300 (Dako, A4502), and CD31 1:100 (Dako, M0823).

**Figure 1:**
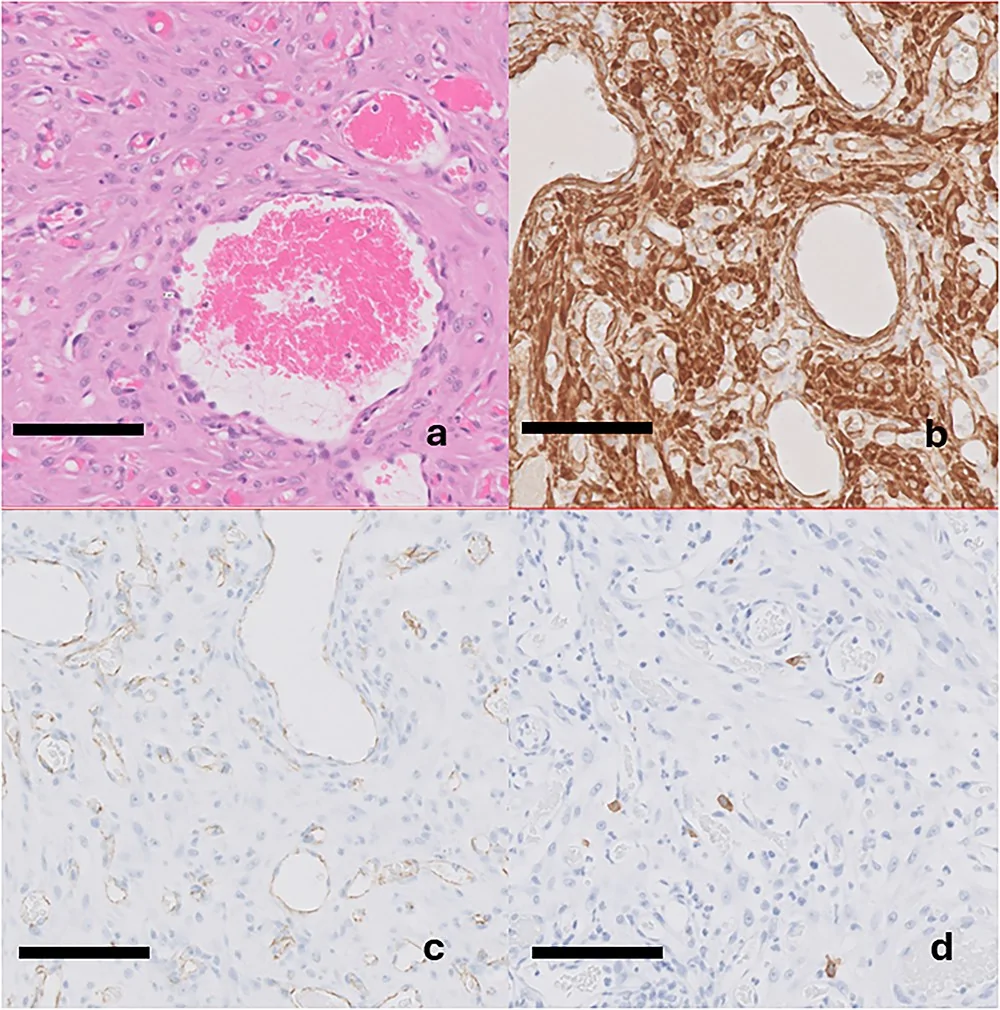
(A) Mass with hematoxylin and eosin staining, showing blood vessels surrounded by densely packed spindle cells. (B) Representative example of immunohistochemical labeling for smooth muscle actin, spindle cells between vessels label positively. (C) Representative example of immunohistochemical labeling for CD31, endothelial cells label positively, spindle cells between vessels are negative. (D) Representative example of immunohistochemical labeling for CD117. Infiltrating mast label positively (brown), the spindle cells are negative. (Scale bar represents 100 μm.)

The atypical cells exhibited diffuse specific marked cytoplasmic labeling with a marker directed against αSMA. Blood vessels within the mass showed labeling with a marker directed against CD31 while the atypical cells did not label with a marker directed against S100 or c-Kit/CD117. Faint immunolabeling with c-Kit/CD117 was associated with a low number of infiltrating mast cells, scattered throughout the mass (Figure 1B-D). Collectively, review of the immunohistochemically labeled slides confirmed the smooth muscle origin of the mass, which was accompanied by a prominent vascular component, thereby establishing a diagnosis of intestinal angioleiomyoma.

Histological results of the lymph nodes revealed diffuse moderate cortical hyperplasia, without evidence of meaningful inflammation or neoplasia. Changes in the liver were consistent with nonspecific vacuolar hepatopathy.

The dog recovered without complications and was discharged the next day. Five months later, during routine follow-up at the referral veterinarian, the CBC was normal (Table S1).

## Discussion

Angioleiomyoma is a rare, benign tumor of smooth muscle origin.^2–5^ It is characteristically associated with an elaborate vascular network, reflecting its purported origin from the tunica media of blood vessels.^5^ In humans, dogs, and cats, most cases of angioleiomyoma are found in cutaneous and subcutaneous tissues.^2^^,^^3^^,^^5^ Infrequently, these tumors can be found in other organs given the ubiquitous nature of blood vessels. In dogs, they are anecdotally described in the nose and nasopharynx,^6^^,^^7^ the spleen,^8^ the orbit,^9^ and the mesentery,^10^ whereas an esophageal angioleiomyosarcoma has been reported in a cat.^11^

Gastrointestinal angioleiomyomas/angioleiomyosarcomas are considered exceptionally rare in humans and are associated with various complications, including intestinal perforation and septic peritonitis, intestinal bleeding and iron deficiency, volvulus and intussusception.^4^^,^^12^^,^^13^ Previously unreported in dogs, the present case was associated with 2 of the complications described above, namely chronic blood loss and, after almost 6 months, intussusception. Severe iron deficiency was the primary instigator for the exhaustive diagnostic work-up in the present case. It was characterized by marked microcytosis and a low transferrin saturation, over many months, without any identifiable source, and despite repeated iron dextran injections on a monthly, and occasionally fortnightly basis. The gastrointestinal tract was considered the most probable route of chronic blood loss, despite the lack of any macroscopic, sonographic or endoscopic evidence to support the suspicion, for several reasons: no other source of blood loss could be found; liver disease had been ruled out as a cause for microcytosis; and occult bleeding to the gastrointestinal tract constitutes a leading cause for iron deficiency in dogs.^1^ Since little additional information was obtained with endoscopic biopsies from the upper gastrointestinal tract, our main differentials included intestinal masses in more distal parts of the intestinal tract, as well as vascular gastrointestinal angiodysplasia/vascular ectasia,^14^ and video capsule endoscopy was considered. However, the need for the procedure was negated once intussusception had developed and angioleiomyoma was diagnosed.

An unusual feature in the present case was failure to diagnose the tumor preoperatively during an abdominal ultrasonography, although 6 months had elapsed between the scan and the final diagnosis, during which the tumor most probably had grown. Intestinal smooth muscle tumors demonstrate variable sonographic changes, ranging from small and focal areas of intramural hypoechoic thickening of the intestinal wall, with or without loss of layering, to larger, discrete, solitary masses with hypoechoic to anechoic areas (corresponding to areas of necrosis).^15^ In veterinary medicine, abdominal ultrasonography is the preferred imaging modality for animals with clinical signs referable to the gastrointestinal tract owing to its widespread commercial availability, lower costs compared to computed tomography (CT), noninvasiveness, and finally, its purportedly high sensitivity for the detection of various intestinal diseases, including intestinal masses.^16^^,^^17^ However, many publications report cases whose diagnosis relied on a positive abdominal ultrasonographic scan, thereby introducing an inherent bias, and describe a heterogenous group of neoplasia. Furthermore, most reports are concerned with malignant gastrointestinal tumors. Correspondingly, reported sensitivities of abdominal ultrasonography for detecting gastrointestinal masses range from 66% to 100%.^18–20^ In humans, CT is preferred over abdominal ultrasonography, owing to increased sensitivity for small lesions, better localization thereof, and increased ability to detect metastases. Additionally, CT overcomes limitations such as intestinal gaseous artifacts that hinder ultrasonography and is less affected by the dog’s size, compared to abdominal ultrasonography.^18^ In cases similar to the one described herein, CT should be considered when gastrointestinal diseases are highly suspected but cannot be demonstrated sonographically.

Another unusual aspect of the present case was the absence of any noticeable, gastrointestinal-related clinical signs during nearly 6 months of follow-up, until intussusception had developed. Similar observations are reported in dogs with gastrointestinal leiomyomas,^16^^,^^21^ with as little as 31% of dogs exhibiting clinical signs in one study.^22^ Therefore, absence of clinical signs such as vomiting, diarrhea, overt gastrointestinal bleeding, or inappetence should not exclude the possibility of gastrointestinal neoplasia.

Histologically, angioleiomyomas are distinguished from leiomyoma by a concomitant interlacing network of blood vessels and are broadly classified in humans into 4 groups depending on their origin: capillary, venous, cavernous, and capillary-venous angioleiomyoma.^5^ Differentiating angioleiomyoma from other smooth muscle neoplasms and perivascular wall tumors, such as glomus tumor, hemangiopericytoma, and angiomyofibroblastoma is based on their histopathological phenotype, and ancillary histochemical and immunohistochemical staining.^4^^,^^16^^,^^17^^,^^22^^,^^23^ Tumors of smooth muscle origin characteristically stain positive with αSMA and inconsistently with desmin.^4^^,^^22^ Ruling out gastrointestinal stromal tumors (GIST) or tumors of neuronal origin, which histologically might resemble leiomyosarcoma, can be assisted by staining for c-Kit and S100 protein, respectively.^16^^,^^17^^,^^23^ In the present case report, the positive staining results for αSMA, lack of reactivity against S100 protein, and the extensive vascular network within the mass, whose endothelial origin was supported by labeling for CD31, corroborated the diagnosis of angioleiomyoma. The scattered, weak immunolabeling with c-Kit/CD117 was the result of low numbers of infiltrating mast cells,^24^^,^^25^ and was not consistent with a GIST.

Criteria for differentiating angioleiomyoma from angioleiomyosarcoma are ill-defined, and rely on the mitotic rate, cellularity, and extent of cellular atypia, degree of anisocytosis/anisokaryosis, and presence of invasive growth patterns and necrosis, or both, which collectively allude to a more aggressive behavior, and were absent herein.^2^ Notwithstanding such distinctions, full excision of malignant angioleiomyosarcomas can confer long, disease-free intervals and can also prove curative,^2^ while conversely, fully excised benign angioleiomyoma can recur.^3^^,^^6^ Furthermore, distant metastases are not reported with either angioleiomyoma or angioleiomyosarcoma in dogs. Taken together, prognosis is considered favorable if full excision is feasible.

In conclusion, intestinal angioleiomyoma should be considered as another differential for intestinal neoplasia in this species. It was associated with severe iron deficiency and anemia as the only abnormality, in the absence of a sonographically discernible mass initially and without any clinical signs referable to the gastrointestinal tract, until intussusception had developed. Definitive diagnosis and classification entail the use of special stains to corroborate the vascular and smooth-muscle origin of the tumor, and to exclude other intestinal neoplasia of neuronal origin. Based on cases of angioleiomyoma in other organs and the dog described herein, surgical excision can be curative, irrespective of histological criteria of malignancy.

## Supplementary Material

supplementary_table_1_aalag281

## Off-label antimicrobial declaration

The authors declare no off-label use of antimicrobials.

## Institutional animal care and use committee or other approval declaration

The authors declare no institutional animal care and use committee or other approval was needed.

## Abbreviations

CT: computed tomography
GIST: gastrointestinal stromal tumor
UIBC: unsaturated iron-binding capacity
αSMA: α-smooth muscle actin
