# Supplementary material for: Intestinal angioleiomyoma in a dog
Source: J Vet Intern Med. 2026 Oct 27;40(5):aalag281. doi: 10.1093/jvimsj/aalag281 (PMC13712304; doi:10.1093/jvimsj/aalag281)
Supplement: Supplementary file 1 [file supplementary_table_1_aalag281.docx]

**Supplementary Table 1**: Selected CBC and chemistry analytes in a dog with severe iron deficiency secondary to intestinal angioleiomyoma.

| Analytes | First visit | 2^nd^ visit* | 3^rd^ visit* | 4th visit* | Reference interval |
| --- | --- | --- | --- | --- | --- |
| Leukocytes (x10^3^/μL) | 21.61 | 13.96 | 23 | 7.9 | 5.05-16.76 |
| Red blood cells (x10^6^/μL) | 6.10 | 7.27 | 7.8 | 6.9 | 5.65 - 8.87 |
| Hemoglobin  (g/dL) | 7.6 | 9 | 9.8 | 17.4 | 13.1 - 20.5 |
| Hematocrit  (%) | 23.5 | 29.5 | 32 | 52.5 | 37.3 - 61.7 |
| MCV  (fL) | 38 .5 | 40.6 | 38.8 | 76 | 61.6 - 73.5 |
| MCHC  (g/dL) | 32.3 | 30.5 | 31 | 33 | 32.0 - 37.9 |
| RDW  (%) | 26.5 | 32.3 | 31 | 14.2 | 13.6 - 21.7 |
| Platelets  (10^3^/µL) | 193 | 360 | 376 | 279 | 148 - 484 |
| Bile acids  (µmol/L) | __ | 3.9 (fasted) 5.2 (post-prandial) | __ | __ | <25 |
| Serum iron (mg/L) | __ | 0.1 | __ | __ | 0.97­2.63 |
| UIBC (mg/L) | __ | 3.87 | __ | __ | 1.83­2.26 |
| TIBC saturation  (%) | __ | 2.5 | __ | __ | 27-66 |

MCHC, mean corpuscular hemoglobin concentration; MCV, mean corpuscular volume; RDW, red blood cell distribution width; TIBC, total iron binding capacity (calculated value, the sum of serum iron+UIBC); UIBC, unsaturated iron binding capacity

*1^st^ visit- upon initial presentation; 2^nd^ visit, after 1 injection of iron dextran; 3^rd^ visit, 6-months after 1^st^ visit, and before surgical removal of the angioleiomyoma; 4^th^ visit- 5 months post-operatively
